# Eclecticism, the Best Conservative Practice

**Published:** 1894-05

**Authors:** Charles F. Allan

**Affiliations:** Newburgh, N. Y.


### ECLECTICISM, THE BEST CONSERVATIVE PRACTICE.[1]

BY DR. CHARLES F. ALLAN, NEWBURGH, N. Y.

THE late World's Columbian Dental Congress, held at Chicago last August, was supposedly a congress, a meeting together, for purposes of professional discussion and education, of the leading dentists of the world; those whose status was the highest and most assured, and those whose dictum as to modes of practice was to be accepted as from the best authority. Such, I say, was supposedly the composition of this widely heralded and much-advertised Congress, and those of you who were present and took part in the discussions know how well this high standard was lived up to.

From one of the chairmen I have heard of a discussion in his Section, that of operative dentistry, in which the position was taken by several dentists of national reputation that amalgam should *never* be used as a filling-material. I have the word of the chairman that this position was not seriously controverted. In the account of the Congress as it appears in the September number of the *Dental Cosmos* this discussion is not mentioned, and I take my statement entirely from the chairman of the Section, who I think is present, and can set me right in case I have overstated. This one-sided discussion was brought up incidentally at a meeting of the Second District Society last October. It occurred to me then that as one of many heresies continually and dogmatically being

---

[1] Read before the New York Odontological Society, February 20, 1894.

proclaimed, and assuming strength, not from anything inherent in the position taken, but solely from lack of opposition and from the force of bald assertion, it was time the position taken by the profession at large was defended, and I felt I would like to take part in it.

This opportunity I have, incidentally, in the paper you have kindly invited me to prepare for you this evening, and my subject, "Eclecticism, the Best Professional Practice," was suggested by the above incident.

Conservative practice, strictly speaking, should indicate and include eclecticism, that is if the greater is to include the lesser; if the great end of the conservation of the teeth means the use of all measures at our disposal for that end. In a word, conservatism in dentistry should apply to the end to be accomplished rather than to the means, and I hope to prove that eclecticism is the best road to that end—is the truest conservatism.

The wonderful fair just concluded at Chicago, of which, as Americans, we are all so justly proud, was in effect an epitome of all the beauty and comfort, all the appropriateness and usefulness for the great end desired, that are possible in our day; and this result was only attainable because of the judicious use of every scientific and industrial discovery. So, likewise, are the present high possibilities of dentistry only attainable, and the fair should convey to us and to all the rest of the world a great lesson, meaning to us that all means and all materials at our disposal should be used for the saving of teeth.

Generally with professional men, especially with dentists, whose work is so varied and appeals to such different sides of our nature, the tendency is, as we add years to our lives, more and more to work in ruts. No one is so fully rounded in professional ability but that he recognizes that in some one or more phases of his daily work he is deficient; possibly to be made up by more than average ability in another direction. Now, it is human to want to do most that which we can do best, and, with a given lesion before us to treat, we will favor that side, unwittingly, perhaps, when it is in the balance as against another mode of practice.

Talking lately with one of the most prominent physicians of your city, he took the position that specialists in medicine—oculists, aurists, etc.—were to a certain extent losing prestige and the confidence of physicians and patients as well, not because of any lack of ability in their special lines, but by reason of the fact that at times they saw so little beyond their own sphere. The general health and well-being of the patient being for the time lost sight of in the zealous treatment of the organ or organs diseased. So it is with dentists, particularly those specialists within a specialty.

Every man before me has seen one or more cases of regulating where the result was perfect, judged by the standard of a symmetrical and properly arranged arch of teeth, but if judged by the probable life of the teeth, their present carious condition, and the lack of comfort and consequent lack of confidence in dentists, all due, partially if not wholly, to the regulating procedure, the operation must be considered a dismal failure. As well, you have all seen most beautifully contoured gold fillings, work which you know has taken hours of most conscientious labor, and in which, indeed, is builded part of the life of the operator, and yet from the overtaxing of the strength of the patient there has been begotten a dislike and dread of our profession that has resulted in a neglect and disregard of the teeth that has been fatal to their preservation.

I could multiply examples from the perfect plate-workers and the wonderful bridge-makers, faultless in their technical work, but in some cases, for that very reason, improperly prejudiced in favor of their own solution of a difficulty, in favor of that which they can do best.

All of which goes to show the great necessity of cultivating eclecticism,—the looking at a problem from all sides, and the having that familiarity with all processes and materials that will make our choice of method a properly discriminating and decisive one. Applied to the illustrations I have given you, it would mean in the regulating case that if proper discrimination had been used the young anæmic girl with structurally poor teeth and a very nervous temperament, would not have been put through the months and perhaps years of pain and worry necessary for mechanically moving her teeth into their new and beautifully regular position. The wished-for result has indeed been accomplished, but at too great a sacrifice. Better by far would it have been to have kept that young person well within the limit of her physical endurance, and by perhaps one or two timely extractions and a little use of the corundum have corrected the worst of the deformity, and by so doing have helped to preserve the teeth, and, as well, the confidence of the patient. I think perhaps that nowhere are we called upon for so much broadness of view, for the weighing in the balance of so many opposing conditions, as in the making up of our decisions in these cases.

The same criticism will apply, in a minor degree perhaps, to the examples I gave of the beautifully contoured gold fillings.

No one appreciates more than I do the value of the high ideals set before us by the lamented Drs. Varney and Webb. They sacrificed their lives to the conscientiousness of their work, and we are their beneficiaries; but I do not want to lose sight of Drs. Dunning, Eleazar Parmley, and the few excellent and conscientious soft-foil fillers of a quarter of a century and more ago, and the man who wants to be fully rounded will take lessons from them, and perhaps venerate one class of operators as much as the other.

Nowhere, in the use of materials is eclecticism called for more than in the use of the different preparations of gold, for in our daily work there is occasion for the use of very many of the different kinds furnished us by the dealers.

The dentist moderately young in the profession, who came in under the glamour cast over our fraternity by the use of cohesive gold, particularly when used in conjunction with the electric or the mechanical mallet, and who was taught by his preceptor and college teachers to ignore altogether soft foil (so-called) and worship and use only cohesive foil, has practised under a great disadvantage to both himself and his patients. A proper eclecticism would have indicated to him, as he saw very frequently in the early years of his practice the wonderful results in the saving of teeth obtained by soft foil, that these results could not have been obtained by chance, and were not the result of chance; and that inherently there were good qualities in the material when properly used. This is, I say, the reasonable deduction to be made by any observing and unprejudiced dentist.

I have no desire to belittle the enormous help and advance the introduction of cohesive foil was to us in the making of gold fillings; indeed it can hardly be overestimated. By its use former ideals were made every-day practical results; a new standard of excellence was indeed raised, and the old flat fillings and parallel-sided spaces, so injurious to the teeth, so ruinous to the gingival borders, and uncomfortable to the patient, were relegated to the past, and were not to be resurrected for any length of time even by the eloquence and ingenious arguments of an Arthur.

But all the same, there is a place in our practice for many other kinds of gold than cohesive foil. The various kinds given us by the dealers have widely different qualities, and a proper eclecticism indicates that we should take advantage of these varying qualities to the utmost.

Without particularizing, as regards the mechanical differences and advantages, the plasticity of non-cohesive foil, and in some degree the very fact that it does not make a homogeneous filling, are valuable points in its favor, and often indicate its use ; and the fact must never be forgotten that a superstructure of cohesive foil can be built upon a substratum of non-cohesive foil with as much absolute certainty of permanence of surface as if the filling were from the foundation built of cohesive gold.

The ideal shape of cavities, with strong walls and margins and but very little undercut, is seldom realized in actual practice, and the ease and perfection with which soft foil adapts itself to irregular surfaces and can be tucked away in undercuts more or less deep, combined with the absence of percussion, occasionally so trying to our patients, all go to show that there are great advantages in the use of non-cohesive foils, and that a proper eclecticism compels us to make use of these advantages.

A form of gold perhaps more unreasonably slighted than any other, considering its great merits, is that which we know of as crystal gold. I say unreasonably slighted, because it is in many cases condemned for faults long since eliminated. In the early years of its manufacture the process used was entirely different from that obtaining now; there was apt to be a certain amount of free acid in the preparation, and fillings made from it were likely to discolor; it was not then the absolutely pure form of gold it is now. At present no charge of impurity can lie against it.

One of your most prominent members told me but a few weeks ago that he had never used it, and he is a very eclectic practitioner. It is the most cohesive form of gold we have, and is that form which can be manipulated with the least pressure; so that for use in moderately small cavities, in teeth very sore from wedging, it is invaluable; and generally, wherever a very cohesive and amenable form of gold is desired before a filling has reached the condition of a smooth level surface, you will find nothing better to your hand than crystal gold.

Most of the gentlemen before me have occasionally, when finishing up a contoured surface of a proximate filling, burnished on one or more pieces of heavy foil, doing so mainly because of the lack of space for plugger-points and rather of necessity, but few of you, I fancy, have considered it a mode of contouring to be generally practised. Some time ago my friend Dr. Van Woert told me he was in the constant habit of using a certain make of No. 30 rolled gold in this manner, and I have since then used it in the same way very frequently. To be able, in contouring a filling in a bicuspid, with the tooth very tender from all kinds of manipulation, and the space commencing to be contracted as the filling is built out,—to be able then to give up the mallet in any of its forms, and gently burnish on the contouring knuckle with a knowledge that it will stand, is a relief to the patient and a comfort to yourself that only has to be stated to be appreciated.

These are salient examples; but the properly eclectic dentist will probably require and use many other kinds of gold than those mentioned. The gold and platinum or gold and iridium in the heavy numbers, for building up molars, where great wear is expected; crystalloid gold for very heavy work because of the rapidity with which it builds up a filling,—these, with velvet and other cylinders, electric and ribbon gold, for occasional use with the electric or mechanical mallets, are all useful and at times necessary.

The most flagrant case—very seldom seen, I am glad to say—of the absence of eclecticism, where prejudice or ignorance masquerade under the guise of conservatism, is in the non-use of amalgam. That there is a place for amalgam in our practice is proved by the fact that it is used to a more or less extent by at least nineteen-twentieths of the profession, and possibly includes ninety-nine out of a hundred. Indeed, the use of this material is so general that I should hardly be called upon to take it into account at all, under my illustrations of non-eclecticism, were it not that this hundredth man was so rampant, persistent, and assertive. He will not down. It seems to be another but very aggravated case of the obstinate eleven jurors.

I am aware that much is sometimes said in the heat of discussion that the speaker would wish to revise or qualify after time has been given him for reflection, and that many assertions are made dogmatically, but with mental reservations that to the speaker seem inherent to his proposition. The young hearer or reader, however, knows nothing of mental reservations or exceptions, and takes the speaker to mean what he says. He hears the dogmatic assertions from men high in the profession, and these assertions are not, or but mildly, controverted; he knows they are contrary to the teachings and practice of his preceptor and college professors, and he naturally wonders whether he belongs to a profession at all. Now, we have a reason for the faith that is in us, and we should always be ready, yea, anxious to give it, and consistency and the integrity of the profession demand that we should. Our profession should not be discredited while we have a voice to prevent it.

Another case for eclecticism is in the use of something else than our ordinary filling-materials for the repair of decayed or broken front teeth; something that from an artistic point of view will be a repair; something that will resemble the tooth, more particularly where the lesion is in the cutting-edge. Corners can be so neatly and strongly replaced with porcelain, and breaks in the middle of the cutting-edge can be so easily repaired by merely cutting a dove-tailed slot and slipping in a previously matched and fitted piece of porcelain, that the free and inartistic display of gold in these places can be and should be avoided.

Turning now from filling-materials to the mechanics necessary for the preparation of cavities and the filling of teeth, we will find probably a greater lack of, and a greater need for, eclecticism than in any other portion of our work. Here, more than in any other department are we apt to work in ruts; we differ so radically in what I may term our mechanical make-up or tendencies, which with many might be called mechanical idiosyncrasies, that there are broad differences between any two of us. In no other department can we learn so continually of our neighbor; in no other branch of our profession is it so necessary for our improvement to be on intimate terms with our dental friends.

Free personal intercourse with other dentists, indeed, is the greatest possible promoter of eclecticism, and in this part of our work is especially desirable.

Most of us can give something to our neighbor, and many of you can give a great deal, and this free intercourse is all in the line of changing a decision from doing that which we can individually do best, to doing that which is actually best for the case in hand. How beautifully ethics and professional gain work in together! The evenings and Sunday afternoons spent with a professional friend should be the most valuable time of the year.

The main importance, I take it, of the various mechanical aids that have been offered us from year to year lies in the direction of simplifying operations; one important phase of which is in making simple and easy cavities out of compound and difficult ones. When we eliminate one difficulty we have so many more resources left to devote to the other difficulties of an operation, and this is the true basis from which to estimate the value of all our mechanical aids.

You have in this great metropolis one or more honored co-laborers who very rarely if ever use the rubber dam; they are men of the highest professional standing, whose ideals are the same as yours; you look up to them for advice and respect them for their results, and yet if the standard I have advocated in this paper is correct they have not fully lived up to the best that is in them.

A dentist who does not use the rubber dam may in some cases equal the best work of him who does, but that will not give the former man a clean bill. The question is not, have I filled the cavity as well as my neighbor? but, have I done it as well and with as much comfort to my patient as if I had used the rubber dam?

*A man's standard should not be that of somebody else, but it should be the best that is in him when he has taken advantage of every aid at his disposal.*

In the use of many therapeutic remedies, as well as in the performance of mechanical operations, the rubber dam is almost a *sine qua non*, and the man who disclaims its help must of necessity be a great loser.

In my own city there are one or two dentists who take pride in the fact that they never use the dental engine,—have none in their offices,—and you will find, I fancy, similar cases in every large city in the land. Most of such people decline the great assistance of proper instruments and machinery to pander improperly and unreasonably to the physical weaknesses of their patients, and it is their patients who in the end suffer most sadly. They use a double-edged sword, and while the temporary makeshifts to which they resort cause the loss of the teeth, their strictures, real or implied, on the bulk of the profession who do use machines and machinery, encourage distrust; this is dishonesty, and does not come within the limits of this paper for discussion.

A charlatan is of necessity a narrow-minded man, and a narrow-minded man, in the very nature of things, cannot be eclectic, for eclecticism goes with and encourages breadth of character and sincerity of purpose, the best elements of professional life.

There are, however, men in our profession of an entirely different class, honest to a degree, who have never been able to see the excess of merit of machines over hand instruments. They are mostly among the elder members of the profession, who, having attained the greatest possible facility in the digital manipulation of hand instruments before dental engines were invented, unwittingly magnify their own dexterity, as opposed to the benefits to be gained by the sometimes use of the dental engine; and to expect them to change their life-long practice is, possibly, hoping for eclecticism under very difficult conditions.

It hardly seems necessary, however, to argue for the use of necessary machinery. The dental engine makes possible some work which is impossible with hand instruments alone, and it makes easy and rapid and endurable much work that would be very difficult and tedious without it. Our capacity for dental work is infinitely extended by its use, and the man who does not take advantage of it can but be a loser. What he can do for his patients under certain circumstances, what *he only* can do best, cannot be what is best for his patients.

The same argument applies to the use of the many devices, such as rubber-dam clamps, separators, etc. The earnest eclectic operator must have them in all their forms; to illustrate, you have a very large cavity, nearly exposing the pulp and encroaching on the gingival borders, on the distal face of a second superior molar, in a mouth very moist and with the muscles rigid; a combination as you all know very difficult to manage. Now, how much it will simplify your operation if you have a rubber-dam clamp so neatly and properly fashioned that it will grasp the third molar without impinging on the festoons of the gum, and so practically without pain; that will stay put exactly as you place it, without any movement, and which has flanges large enough to hold a napkin in place if necessary, and will of course keep the rubber dam in position if used. More than half the battle is at once won. Such clamps are to be had, but I do not think they are generally used, though I know the want of a properly constructed clamp for superior third and second molars has been seriously felt for many years.

Now, if my arguments have been founded on correct theories and a proper standard of ethics, and if my illustrations have been of any pertinency to enforce my arguments, surely the absolute necessity of eclecticism to bring out what is best in us professionally is evident. I almost think I hear some of you whisper, "This goes without saying,"—in theory perhaps, but how is it in practice?

When men prominent in the profession, before the World's Columbian Dental Congress, say that amalgam should *never* be used; when others in various places say that cohesive gold-foil is the only form of gold proper to be used, which statement is further supplemented by one from others still more radical, who affirm that it should only be used in connection with the electric mallet; when some dentists say they never use the rubber dam, and your neighbor asserts he never uses the dental engine, you will pardon me if I say before this, the most eclectic and progressive dental society in the world, that I believe a plea, and an urgent one, for eclecticism, is in order.

